# Removal of Small Kernels Reduces the Content of *Fusarium* Mycotoxins in Oat Grain

**DOI:** 10.3390/toxins12050346

**Published:** 2020-05-23

**Authors:** Guro Brodal, Heidi Udnes Aamot, Marit Almvik, Ingerd Skow Hofgaard

**Affiliations:** Norwegian Institute of Bioeconomy Research (NIBIO), P.O.Box 115, N-1431 Ås, Norway; heidi.udnes.aamot@nibio.no (H.U.A.); marit.almvik@nibio.no (M.A.); ingerd.hofgaard@nibio.no (I.S.H.)

**Keywords:** T-2 toxin, HT-2 toxin, deoxynivalenol (DON), enniatin B (EnnB), size sorting, unprocessed cereals

## Abstract

Cereal grain contaminated by *Fusarium* mycotoxins is undesirable in food and feed because of the harmful health effects of the mycotoxins in humans and animals. Reduction of mycotoxin content in grain by cleaning and size sorting has mainly been studied in wheat. We investigated whether the removal of small kernels by size sorting could be a method to reduce the content of mycotoxins in oat grain. Samples from 24 Norwegian mycotoxin-contaminated grain lots (14 from 2015 and 10 from 2018) were sorted by a laboratory sieve (sieve size 2.2 mm) into large and small kernel fractions and, in addition to unsorted grain samples, analyzed with LC-MS-MS for quantification of 10 mycotoxins. By removing the small kernel fraction (on average 15% and 21% of the weight of the samples from the two years, respectively), the mean concentrations of HT-2+T-2 toxins were reduced by 56% (from 745 to 328 µg/kg) in the 2015 samples and by 32% (from 178 to 121 µg/kg) in the 2018 samples. Deoxynivalenol (DON) was reduced by 24% (from 191 to 145 µg/kg) in the 2018 samples, and enniatin B (EnnB) by 44% (from 1059 to 594 µg/kg) in the 2015 samples. Despite low levels, our analyses showed a trend towards reduced content of DON, ADON, NIV, EnnA, EnnA1, EnnB1 and BEA after removing the small kernel fraction in samples from 2015. For several of the mycotoxins, the concentrations were considerably higher in the small kernel fraction compared to unsorted grain. Our results demonstrate that the level of mycotoxins in unprocessed oat grain can be reduced by removing small kernels. We assume that our study is the first report on the effect of size sorting on the content of enniatins (Enns), NIV and BEA in oat grains.

## 1. Introduction

Several species of the fungal genera *Fusarium* are common pathogens of small grain cereals. *Fusarium* spp. infect and cause damage to the head and grain of cereals, especially under moist conditions. The disease, known as Fusarium Head Blight (FHB), is one of the most important diseases in wheat (*Triticum aestivum*), oats (*Avena sativa*) and barley (*Hordeum vulgare*). During development and maturation of infected heads, *Fusarium* species can produce several mycotoxins which can lead to severe contamination of grain [1]. Mycotoxin-contaminated grains do not necessarily show disease symptoms which makes them difficult to identify. Consumption of grain and grain-based products containing *Fusarium* mycotoxins can cause many harmful health effects in humans and animals, and *Fusarium* toxins are therefore one of the most important quality and safety risks of cereal grain for food and feed [2,3,4]. In addition, workers at grain elevators and mills may be exposed to mycotoxins by inhalation and skin permeation of grain dust during grain processing [5]. To reduce the risk, the European Union (EU) has set maximum levels for some mycotoxins in cereal grain and cereal-based food products for human consumption, and has recommended guidance values for its content in animal feed [6,7].

The mycotoxin deoxynivalenol (DON) is common and frequently occurs in oat grains [8,9,10,11]. Moreover, the HT-2 toxin and T-2 toxin are often found more frequently in oats than in other cereal species, and sometimes at high concentrations [9,10,12,13,14,15]. HT-2 and T-2 toxins are closely related (HT-2 is the deacetylated form of T-2), often occur together [12] and their occurrence concentrations are often considered together as a sum of HT-2 and T-2. Throughout this study we use the denomination HT2+T2 for the sum of these toxins. In addition to DON and HT2+T2 toxins, zearalenone (ZEA) and several unregulated mycotoxins such as nivalenol (NIV), enniatin A (EnnA), enniatin A1 (EnnA1), enniatin B (EnnB), enniatin B1 (EnnB1) and beauvericin (BEA) often occur in oat grains [8,9,10,11]. In Norway, 3-acetyl-deoxynivalenol (3-ADON) is the dominating acetylated chemotype, although 15-acetyl-deoxynivalenol (15-ADON) has been detected [16].

Besides the importance of oats as a raw material for animal feed concentrate (compound feed), oats grown for human consumption have increased during the last few years due to their beneficial nutritive properties [17]. On the other hand, it has been reported that consumers with a high relative intake of cereals compared to their body weight have a HT2+T2 and a DON exposure that may exceed the Tolerable Daily Intake (TDI) [18]. HT2+T2 toxins are considerably more toxic than DON (TDI 0.02 and 1.0 µg/kg body weight/day, respectively) [19]. The maximum levels for DON and ZEA in unprocessed oat grain for food are 1750 and 100 µg/kg, respectively [6]. However, no regulated maximum levels have been set so far for HT2+T2 in cereals and cereal products, but the European Commission has recommended an indicative level of 1000 µg/kg for HT2+T2 in unprocessed oats [20]. When concentrations of HT2+T2 are detected above this level, the EU member states should perform investigations to identify factors resulting in these levels and investigate the effect of feed and food processing on the presence of these toxins.

In oats, the largest proportion of the mycotoxins is located in the hulls. Thus, de-hulling, i.e., removing hulls (glumes and husk) from the kernels before further processing into oat flakes and other products is an efficient method to reduce the mycotoxin content of oats. Commercial processing of oats has been reported to reduce the content of DON, T2 and HT2 by 80%–95%, with the major loss occurring during de-hulling [21,22,23,24,25]. However, de-hulling is part of the processing of cereal grain. On the other hand, cleaning and size-sorting of raw grain, normally performed as a first step to remove dust, weed seeds, chaff/straw pieces and small, lightweight and damaged kernels before further processing, is accepted according to European legislation, to be carried out on unprocessed grains [6,20]. Several studies have shown that cleaning and sorting of cereal grain can reduce the content of mycotoxins, although variable effects have been reported. Most data are available for reduction of DON in wheat, but also other *Fusarium* toxins, e.g., HT2+T2, NIV and ZEA have been analyzed. The effects of cleaning and sorting by various methods and procedures on the reduction of mycotoxins in wheat, as well as in a few studies in barley and oats, have been reported to vary from no reduction to up to more than an 80% reduction and have even been reported to increase levels in a few cases [3,26,27,28]. The effect of removing the small grain fractions by size sorting, i.e., after separating the kernels on sieves according to kernel size, varies depending on the sieve sizes used. Despite different degrees of mycotoxin reduction reported by the cleaning and sorting of grains, overall results indicate that these operations may efficiently reduce the mycotoxin levels in highly contaminated cereals before further use/processing.

Data on the effects of removing small grain kernels on the mycotoxin content in oats are limited. The aim of the present study was therefore to investigate to what extent the removal of small kernels can contribute to the reduction of the mycotoxin content in oat grains and thereby improve the quality of the remaining grain. Our hypothesis was that it is feasible/achievable to reduce the mycotoxin content in raw oat grain by size sorting/cleaning out the smaller kernels. Grain from 24 Norwegian oat grain lots (14 from 2015 and 10 from 2018) were sorted into a large and a small kernel fractions by passing the grain through a 2.2 mm laboratory sieve. The large and small kernel samples, in addition to samples of unsorted grain, were analyzed for content of HT2 and T2 (reported together as HT2+T2), DON, ADON (3- and 15-acetyl-deoxynivalenol analyzed together), NIV, EnnA, EnnA1, EnnB, EnnB1, BEA and ZEA. The mycotoxin concentrations in the two grain fractions were compared with the concentrations in the unsorted grain.

## 2. Results and Discussion

### 2.1. Mycotoxin Content in the Oat Grain Lots

**Figure 1:**
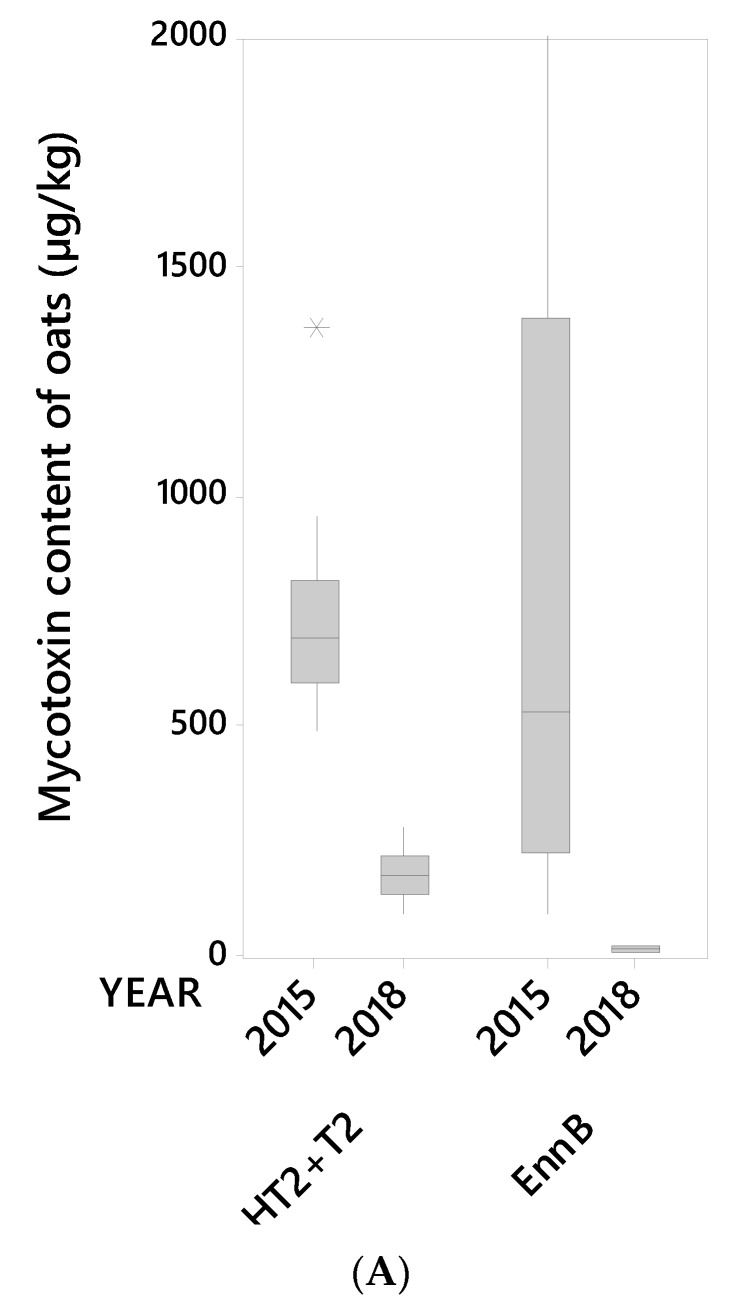

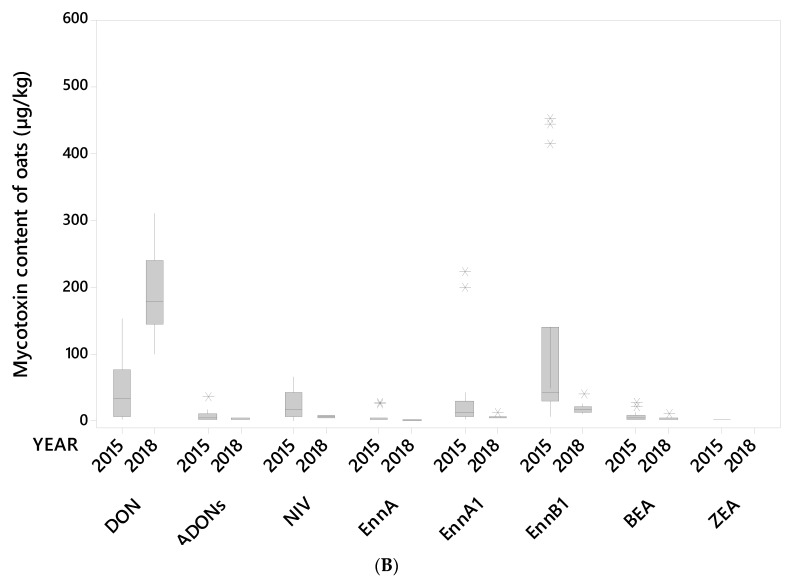
(**A**) Median, interquartile range, range and outlier (*) for the content (µg/kg) of the mycotoxins HT2+T2 and EnnB (Enniatin B) in 14 oat grain lots from 2015 and 10 oat grain lots from 2018 (unsorted samples). (**B**) Median, interquartile range, and outliers (*) for the content (µg/kg) of the mycotoxins DON (deoxynivalenol), ADON (3- and 15-acetyl-deoxynivalenol), NIV (nivalenol), EnnA (enniatin A), EnnA1 (enniatin A1), EnnB1 (enniatin B1), BEA (beauvericin) and ZEA (zearalenone) in 14 oat grain lots from 2015 and 10 oat grain lots from 2018 (unsorted samples).

**Figure 2:**
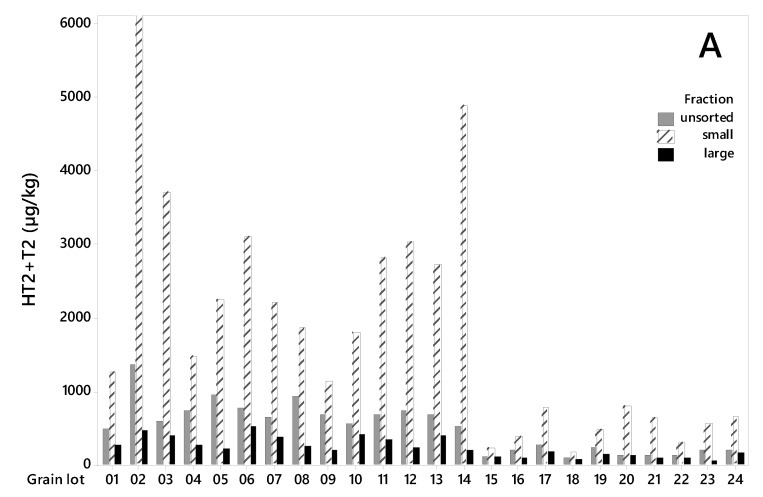

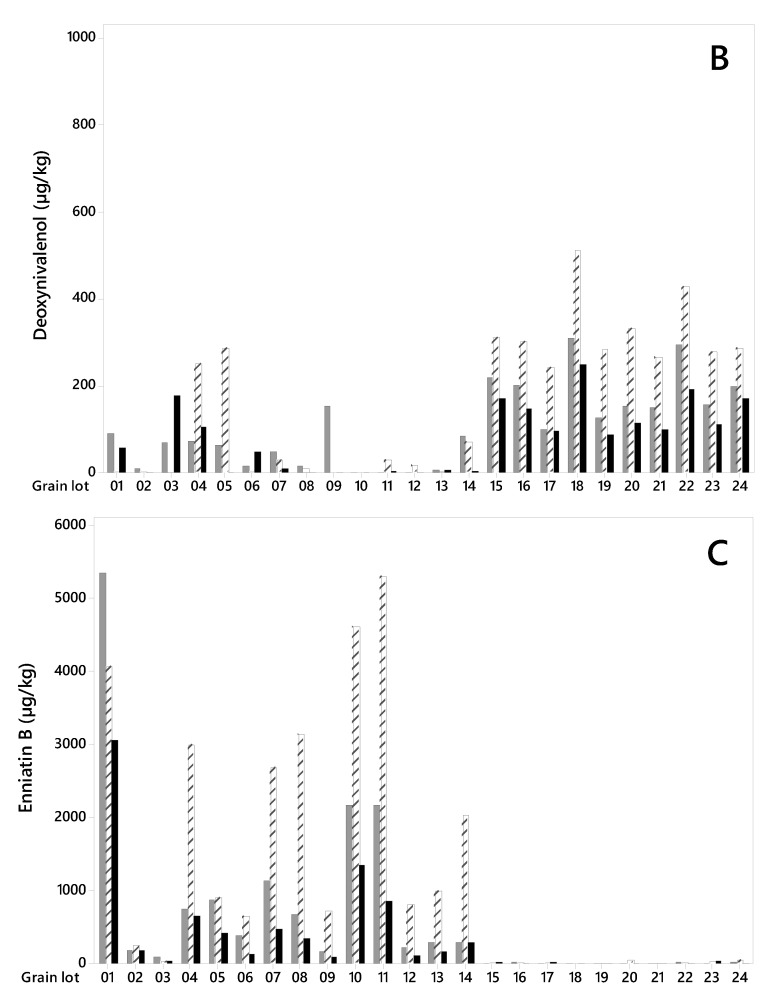
Concentration levels (µg/kg) of HT2+T2 toxins (**A**), deoxynivalenol (**B**) and Enniatin B (**C**) in 24 unsorted oat grain lots and in large and small kernel fractions after size sorting on sieve size 2.2 mm. Lot 1–14 from 2015, lot 15–24 from 2018. Note the different values on the concentration level axes.

A visual summary of the concentrations of the ten mycotoxins detected in the 24 oat grain lots (unsorted grain) is shown in Figure 1A,B. Most grain lots contained all the tested mycotoxins. Moderate to high mycotoxin levels in unsorted grain samples were detected for HT2+T2 toxins and EnnB in 2015, and DON in 2018 (Figure 2). Similar contrasting occurrences of HT2+T2 vs. DON in oats have been reported in other studies [9,29,30]. The mycotoxin levels in samples of unsorted grain of the remaining mycotoxins, i.e., ADON, NIV, EnnA, EnnA1, EnnB1, BEA and ZEA were generally low in samples from both years. ZEA was only detected in 2015 samples. Our results are in line with other reports on occurrences of mycotoxins in Norwegian oat grains [9,12,25,31].

### 2.2. Mycotoxin Content in Unsorted and in Large Kernel Fraction of Oats

#### 2.2.1. HT2+T2

**Figure 3:**
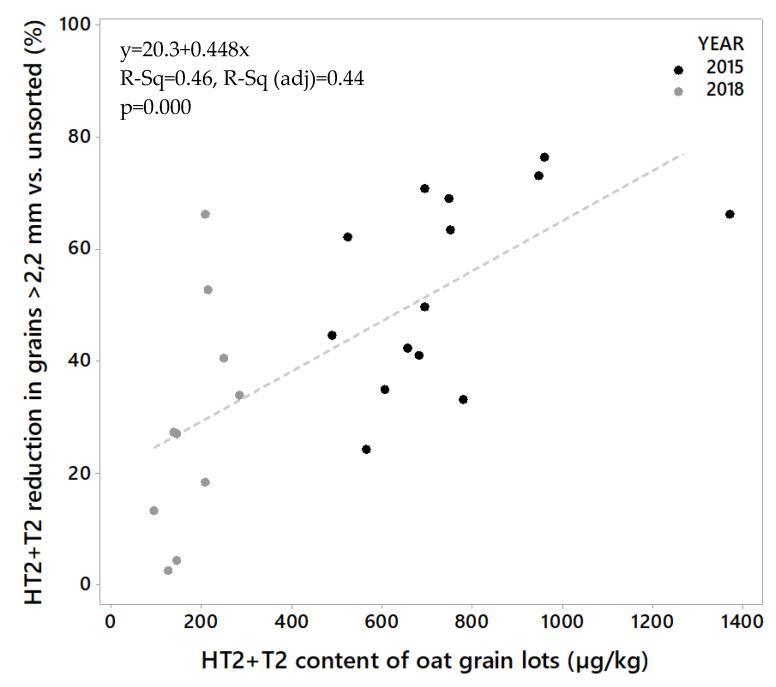
Percentage reduction of HT2+T2 toxins in oat grain samples from 2015 and 2018 in large kernel fraction (after removing small kernel fraction by sieve size = 2.2 mm) vs. concentration level in grain lots (unsorted grain).

All grain lots contained HT2+T2 toxins (Figure 2A). The levels were considerably higher in the grain from 2015 (lot 1–14) than in the grain from 2018 (lot 15–24). The HT2+T2 concentrations in unsorted grain varied from 486 to 1368 µg/kg (mean 745 µg/kg) among the samples from 2015, and from 92 to 282 µg/kg (mean 178 µg/kg) among the samples from 2018 (Table 1). After sorting, we detected significantly lower HT2+T2 in the large kernel fractions than in the unsorted grain. The concentrations in the large kernel fraction varied from 197 to 522 µg/kg (mean 328 µg/kg) among the samples from 2015, and from 70 to 187 µg/kg (mean 121 µg/kg) among the samples from 2018. In 2015, this corresponds to an average reduction in HT2+T2 concentration of 56% (varying from 24% in lot 10 to 76% in lot 5) in the large kernels compared to the unsorted grain. In 2018, the average reduction in HT2+T2 concentration was 32% (varying from only 2% in lot 15 to 66% in lot 23) (Table 1). The average weight reduction after removal of the small grain fraction was 15% and 21% of the weight of the samples from the two years respectively (Table 2). We did not observe any relationship between the percentage weight reduction and percentage of HT2+T2 reduction (*R*^2^ = 0.00, *p* = 0.919), or between weight reduction and HT2+T2 levels in unsorted samples (*R*^2^ = 0.04, *p* = 0.328) when calculated for all 24 grain lots together. However, a significant relationship between percentage weight reduction and HT2+T2 levels was observed (*R*^2^ = 0.47, *p* = 0.010) for samples from 2015, by omitting the highest contaminated seed lot (No. 2). Only a few studies on the effects of removing small grain kernels on HT2+T2 in oats by size sorting have been found. A Swedish study reported markedly reduced concentrations of HT2+T2 (not quantified) after removing the kernels that passed through a sieve size of 2 mm [32]. A study in Finland, also using a sieve size of 2 mm obtained around a 30%–35% reduction [33], which agrees with our results from 2018 samples and is somewhat lower than what we obtained from the 2015 samples. It was interesting to observe that the effect of removing small kernels was considerably higher in the oats with relatively high HT2+T2 levels (mean 745 µg/kg, 2015 samples) than in oats with lower levels (mean 178 µg/kg, 2018 samples). The extent of toxin reduction increased with toxin levels, and linear regression showed that this relationship was significant (*p* = 0.000, *R*^2^ = 0.44, Figure 3). Moreover, a few other studies have reported the highest reduction of HT2+T2 in the highest contaminated oats by size sorting and de-hulling [21,32].

As in our study, large differences between samples on the effect on HT2+T2 levels in oats by cleaning/sieving (laboratory-scale grain cleaner, sieve size 1.75 × 20 mm) were reported in a German study [22]. They observed reductions in the range from 0% to 100%, which is even more inconsistent than our data. In a study of barley, the content of HT2 was reduced by 68% and T2 by 81% on average, after around 13% of the sample was removed by using a 2.5 mm sieve [34]. In durum wheat, 54% reduction in the HT2+T2 concentration was observed after a vigorous cleaning procedure (aspiration and two sieves: 5 × 15 mm and 2 × 19 mm) [35]. Concentration levels of HT2+T2 in oats have been reported to be higher in rachis and glumes than in kernels [36]. As small kernels contain a higher proportion of glumes and pericarp than large kernels, removing small kernels will contribute to a reduced mycotoxin content. Our results and other reported data imply that cleaning and size sorting can be useful methods to reduce the concentrations of HT2+T2 in unprocessed grain of oats and other small grain cereals, although the effect will vary among grain lots.

#### 2.2.2. DON

DON was detected in all grain lots (Figure 2B), however its concentration levels were higher in the grain from 2018 (lot 15–24) than in the grain from 2015 (lot 1–14). The concentration levels varied from 100 to 309 µg/kg (mean 191 µg/kg) in unsorted samples from 2018 and from quantification limit (LOQ = 1 µg/kg) to 153 µg/kg (mean 46 µg/kg) in samples from 2015 (Table 1). For the grain harvested in 2018, removing the small kernels resulted in significantly lower DON concentrations in the large kernels (varying from 89 µg/kg to 249 µg/kg, mean 145 µg/kg) compared to the unsorted grain. On average, we detected 24% less DON in the large kernels in 2018 samples, however, the reduction varied from only 3% (lot 17) to up to 35% (lot 22). Despite low DON levels in the 2015 samples, we observed a trend towards a lower mean concentration in large kernel fractions (30 µg/kg) compared to unsorted grain (46 µg/kg) (Table 1). However, the effect of removing the small kernels varied from a reduction to an increase in DON content, and no significant difference in DON levels was detected between the grain fractions.

Limited data exist on the size-sorting effects on DON in oats. A Finnish study observed a 30%–40% reduction in DON concentrations using a 2 mm sieve [33], which is somewhat higher than the 24% reduction we obtained in our 2018 samples. Size sorting of barley in the same study resulted in around a 50% lower content of DON in large grain. Another study in barley reported an 80% reduction of DON after removing the small kernels by using a sieve size of 2.5 mm, however, the effect differed between cultivars [34]. Several studies in wheat, using different sieve sizes, aspiration and cleaning technologies resulting in large variations in the amounts of by-products (e.g., waste, screenings, offals, dockage, pellets etc.) have reported from no and up more than 80% reduction in DON concentrations by cleaning and sorting, but also increased levels have been observed in a few cases [26,27,28]. For industrial cleaning, an expected reduction rate of 20% has been suggested for DON in wheat [37], which is near the 24% reduction we detected in oats by removing the small kernel fraction in the 2018 materials. Our result supports the previous finding that removing small kernels can reduce the concentration of DON in oats, however as with other small grain cereal species, the effect is likely to vary among grain lots.

#### 2.2.3. Enniatins (Enns) and BEA

The prevalence and concentration levels of Enns and BEA in our grain lots were in accordance with previous studies from Nordic countries where these toxins have been reported as common contaminants of cereals occurring generally at low concentration levels. However, they occur occasionally at high levels, and often EnnB is the most common [8,9,10,25,31].

All grain lots contained EnnB (Figure 2C). The concentration levels in unsorted samples were considerably higher in most grain lots from 2015 than in grain from 2018, ranging from 92 to 5356 µg/kg (mean 1059 µg/kg), and from 8 to 25 µg/kg (mean 15 µg/kg), for the two years respectively. After removing the small kernel fraction, the EnnB concentrations in large kernel samples from 2015 was significantly lower than in unsorted grain and varied between 48 and 3064 µg/kg (mean 594 µg/kg) (Table 1). On average, this represents 44% less EnnB content than in unsorted grain. However, the reduction varied between 5% (lot 14) and 63% (lot 6), and in lot 2 we recorded an increase of 2%. For samples from 2018, no reduction in the mean EnnB concentrations was detected after size sorting. EnnB1 was detected in all grain lots. In samples from 2015, the concentrations ranged from 6 to 452 µg/kg (mean 120 µg/kg), and a trend towards lower EnnB1 concentrations in large grain (mean 62 µg/kg) compared to unsorted was observed (Table 1). The EnnB1 concentrations in samples from 2018 ranged from 9 to 38 µg/kg (mean 18 µg/kg), with no difference in the content between large and unsorted grain. EnnA and EnnA1 were also detected in all unsorted grain samples, however, the levels were low, especially in samples from 2018 (Table 1). In samples from 2015, a trend towards a lower content of EnnA and EnnA1 in the large grains compared to unsorted grain was observed. BEA occurred at very low levels in all unsorted grain lots, however, a trend towards a reduction was observed after removing the small kernel fraction in samples from 2015 (Table 1). We assume that our study is the first report on the effect of size sorting on the content of Enns and BEA in cereal grains, as we did not find any published data on this. However, a study of Enns in milling fractions of wheat reported that approximately 40% remained in the final wheat flour, compared to whole grain [38].

Enns and BEA have shown cytotoxic, genotoxic and immunomodulating effects, as well as toxic effects on reproductive systems [39,40]. These toxins have been reported to accumulate in animal tissues and eggs [41]. In 2014, the European Food Safety Authority (EFSA) concluded that acute exposure to Enns and BEA do not indicate concern for human health, but chronic exposure might be of concern [42]. However, due to a lack of relevant in vivo toxicity data, a human risk assessment could not be performed. So far, maximum levels for content of these mycotoxins in food and feed have not been established and at present there is no regulatory requirement to consider or reduce the contamination of Enns and BEA in cereal grains. Our results indicate than EnnB can be substantially reduced in oats by removing small kernels, although no consistent effect in relation to concentration levels was found.

#### 2.2.4. NIV, ADON and ZEA

NIV was detected in all but two grain lots from 2015 and in all 10 lots from 2018, however, the overall concentration levels were low (Figure 1B). After removing the small kernel fraction, NIV was detected in all large kernel samples from 2018, but only in 8 of the 14 large kernel samples from 2015. Despite the low levels, a trend towards lower content of NIV in the large grains (mean 11 µg/kg) compared to unsorted grains (mean 23 µg/kg), was observed in samples from 2015 (Table 1). No published data on the effect of sorting and cleaning on the NIV content in oats have been found, however, what is almost an elimination (> 98%) of NIV during oat processing has been reported [21]. In barley, 94% less NIV were reported after removing the small kernels using sieve size 2.5 mm [43]. In wheat, from below a 10% to around an 80% reduction in NIV by cleaning and size sorting has been reported [28]. Based on our limited results and the literature on the effect on NIV in other cereal species, we assume that removal of small kernels can reduce the NIV content in oat grain.

In this study 3- and 15-ADON were analyzed together as ADON. Although 15-ADON is detected in Norway, 3-ADON is the most common chemotype [16]. ADON was detected in all 24 grain lots. However, the overall concentration levels were low (Figure 1B), ranging from 1 (= LOQ) to 35 µg/kg (mean 8 µg/kg) in the samples from 2015, and from 1 to 4 µg/kg (mean 2.2 µg/kg) in the samples from 2018 (Table 1). After removing the small kernel fraction, ADON was still detected in all large kernel samples from 2018 (mean 1.6 µg/kg), and the level was significantly lower than in the unsorted samples. In 2015, ADON could not be detected in five samples after removal of small kernels. Although the average level (5 µg/kg) in the large kernel fraction was lower than in the unsorted grain, this was not statistically significant. A recent Norwegian study on the distribution of mycotoxins in oat grain reported around a 90% reduction of 3-ADON by de-hulling of grain containing relatively high levels of 3-ADON (mean 485 µg/kg) [25]. Based on that study, together with our limited data, it is reasonable to believe that the content of ADON can be reduced by removing small oat kernels.

ZEA was detected at very low levels (close to LOQ = 1 µg/kg) in 12 of the 14 unsorted oat grain samples from 2015, and no ZEA was detected in the 2018 samples (Table 1). Studies in wheat have found ZEA to be mainly concentrated in the outer tissues of the grain, however, the reduction of ZEA content by cleaning and processing has been reported to vary from a few to up to around 40% [43,44,45]. As no data have been found on the effect of size sorting on the level of ZEA in oats, and because we only detected very low levels in our study, it is not possible to conclude on the effect of size sorting on ZEA in oats.

### 2.3. Mycotoxin Content in the Small Kernel Fraction

Since most mycotoxins are mainly concentrated in the small kernel fractions, in the hulls and in the outer tissues of the grains [21,24,26,44] cleaning, size sorting, de-hulling and further processing will increase the mycotoxin concentrations in the by-products (e.g., screenings, offals, dockage, pellets, bran etc.) [3] In our study, the mean concentration of HT2+T2 in the small kernel fraction was 272% higher for the 2015 samples, and 187% higher in the 2018 samples, compared to the unsorted grain (Table 1). The increase in single samples varied from below 100% to up to 840%, which is the same magnitude that has been measured for HT2+T2 in oat by-products from other size sorting and cleaning studies [21,24,32]. The DON concentrations in our samples were generally low, and despite a mean increase of 71% (ranged from 42% to 145%) in the small grain fraction in samples from 2018, the levels were still moderate (Table 1). A high accumulation (ten-fold) of DON in the offals after cleaning was reported in oats in a Polish study [46]. In our study, the mean concentration of EnnB in the small kernel fraction was 98% and 120% higher in samples from 2015 and 2018, respectively, compared to the unsorted grain (Table 1). However, the concentrations in the small kernel samples varied considerably from a reduction of 60% to an increase of 568%. Higher EnnB1 concentrations were found in the small grain fraction compared to unsorted grain in samples from 2018, representing an increase of 89% (Table 1), whereas in samples from 2015, a trend towards higher mean EnnB1 concentrations in small grains compared to unsorted grains was observed. Except for a study reporting a considerably higher content of EnnB (200% and 375% in shorts and bran respectively) and EnnB1 (around 240% and 300% in shorts and bran respectively) after milling of wheat, no other data have been found on the distribution of Enns in cereal grain fractions [38]. Despite low ADON levels, a significant increase was detected in the small kernel fraction compared to unsorted grain for 2018 samples (Table 1). The by-product fractions are commonly used as raw materials for animal feed. It is important for feed producers to be aware of the risk of extensive increases in the mycotoxin content in by-products. To manage the mycotoxin risk and to decide what to be done with potentially contaminated by-products, proper sampling and analysis are necessary. Based on the contamination level, an evaluation of the economic value of the by-products and the carry-over potential of the mycotoxins, a decision on the inclusion level of feed ingredients can be made [3]. In our study, the highest measured HT2+T2 concentration in the small kernels fraction was 6427 µg/kg (lot No. 2) and 4889 µg/kg (lot No. 14) which would have been of concern if it had been used in feed production.

### 2.4. Conditions Influencing on the Effect of Grain Size Sorting

We observed variable effects of size sorting on the content of HT2+T2, DON and EnnB among the grain lots (Figure 2). One reason for this variation can be the diversity in cultivars (Table 2) which is likely to differ in resistance to *Fusarium* infections and mycotoxin development. In addition, our grain lots originated from two different years and partly from different locations. The degree of *Fusarium* mycelium growth into the kernels and mycotoxin development varies between *Fusarium* species and are in addition to host plant resistance against infection, influenced by cultivation conditions and local weather during the susceptible stages of the host plant and during grain-filling and maturation stages [36,47,48]. This can result in a different distribution of the different mycotoxins in kernels and therefore likely contribute to the different effects of size sorting among the samples in our study. Moreover, cultivar differences in phenological kernel traits itself, such as kernel size probably also contributed to the variation in size-sorting effects. Grain materials for this study were not selected to allow for an examination of the influence of cultivar or location on the size-sorting effect. One important reason for the variation between different studies in the effect of cleaning and size sorting on the mycotoxin content in grain is in the use of different sorting and cleaning methods/technologies, e.g., different sieve sizes and machinery settings, and some studies have included a pre-cleaning step without or with aspiration with varying fan speeds to remove dust, broken grain and other debris. By removing some of the “waste products” prior to sorting, less effects will be obtained by further cleaning and size sorting. This diversity in method will also result in large differences in volume and weight proportions removed in the pre-processing stages e.g., [21,34,44].

### 2.5. Grain Weight Reduction by Size Sorting and Mass Balance Calculations

By passing the raw grain samples through a laboratory sieve (sieve size 2.2 mm) and removing the small kernel fraction, the grain weight was in average reduced by 15% and 21% for samples from 2015 and 2018, respectively (Table 2), i.e., a larger proportion passed through the sieve in the samples from 2018 than in the samples from 2015, indicating generally smaller kernels in 2018 than in 2015. However, the weight reduction varied between 6% and 33% among the 14 samples from 2015, and between 9% and 32% among the 10 samples from 2018. No data was found in the literature on the weight reduction by cleaning or size sorting in oats, however, dehulling has been reported to remove around 30% to 40% of the whole grain weight [21,25]. In a study of barley, the weight reduction varied between 6% and 25% among 15 samples of different cultivars when grain passed a sieve size of 2.5 mm [34].

**Figure 4:**
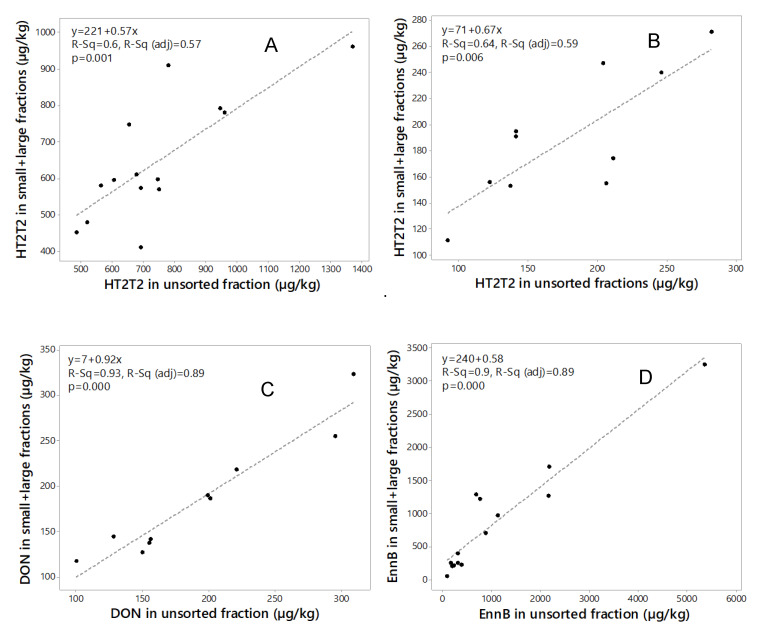
Regression analysis of the sum of toxin in large and small kernel fractions compared to the unsorted fraction for (**A**) HT2+T2 in 2015 (*n* = 14); (**B**) HT2+T2 in 2018 (*n* = 10); (**C**) deoxynivalenol (DON) in 2018 (*n* = 10); and (**D**) enniatin B (EnnB) in 2015 (*n* = 14).

Mass balance calculation of mycotoxin concentrations in unsorted grain and in the sum of the size fractions is an important quality control tool [44]. Mass balance calculated for amounts of HT2+T2 (2015, 2018), DON (2018) and EnnB (2015) in the two fractions from the mycotoxin concentrations and the weight of each fraction, and the sum was compared to the mycotoxin content in the unsorted sample (Table 3). For HT2+T2, the recovery in the sum of the two fractions compared to unsorted grain ranged between 59% and 120% (mean 87%) for 2015 samples and between 75% and 138% (mean 105%) for 2018 samples. For DON, the recovery in the sum of the two fractions ranged between 85% and 117% (mean 96%). The recovery of EnnB in the sum of the two fractions ranged between 51% and 188% (mean 77%). Regression analysis of the mycotoxin amounts in the unsorted grain and the sum amounts in the two fractions (Figure 4) indicated rather a good relationship for DON (*R*^2^ = 0.93, 2018 samples) and EnnB (*R*^2^ = 0.90, 2015 samples), however, some variation in the recovery among the samples were observed for HT2+T2 in samples from both years (*R*^2^ = 0.60 and *R*^2^ = 0.64, for 2015 and 2018 samples respectively). This indicates that the analysis was somewhat inaccurate.

Reasons for some of the discrepancies between the sum of the two fractions and the amounts in the unsorted sample may be due to inaccuracies in sampling, including sample preparation before analysis, the drawing of a small ground sample (5 g) for analysis, and the recovery of the analysis method itself. Sampling is a major source of error in monitoring mycotoxins in cereal grains. It is difficult to obtain homogenous samples partly due to an uneven distribution of mycotoxins within a grain lot [49]. If a sample is not representative it can cause an over- or under- estimation of mycotoxin contamination, and this might have contributed to the variable effects on the mycotoxin content we obtained by size sorting. The type of grinder as well as the method for dividing can influence on the heterogeneity of mycotoxins in a sample. The grinder and sieve size (1 mm) used in our study gave what was perhaps a higher heterogeneity due to a higher particle size than what is optimal [50]. Moreover, small samples with low mycotoxin levels can cause considerable measurement uncertainty.

## 3. Conclusions

Our study showed that by removing the small kernel fraction from the grain, representing on average 15% and 21% of the weight of the samples from the two years respectively, the content of *Fusarium* mycotoxins was considerably reduced. The most notable effects were seen on the concentrations of T2+HT2 toxins, which were reduced by 56% and 32% on average for samples from 2015 and 2018 respectively, and EnnB, which was reduced by 44% in grain lots from 2015. We also observed a clear reduction, on average 24%, in the DON concentrations in the 2018 samples. Moreover, despite low levels, our analyses showed a trend towards reduced content of DON, ADON, NIV, EnnA, EnnA1, EnnB1 and BEA after removing the small kernel fraction in samples from 2015.

For HT2+T2, the reduction obtained by sorting increased with the mycotoxin levels of unsorted grain lots. Ours and other studies experienced variable effects on the mycotoxin content by size sorting, however, removing the small kernel fraction in oats can be a useful method to reduce the mycotoxin contamination. Grain lots are still defined as unprocessed after cleaning and size sorting. Thus, by performing these operations, the grain industry may safely utilize a higher number of unprocessed oat grain lots for further processing in the food and feed chain. Knowledge about the different content of mycotoxins in various grain size fractions increases the possibility of better utilization of oat grains for food and feed and can help to identify grain lots at risk. Because of variable effects, it is also important to analyze the mycotoxin content after size sorting. For several of the mycotoxins, the concentrations were considerably higher in the small kernel fraction compared to unsorted grain. We assume that our study is the first report on the effect of size sorting on the content of Enns, NIV and BEA in oat grains.

## 4. Materials and Methods

### 4.1. Oat Grain Materials

Samples (approximately 0.5 kg, 14% moisture) from 24 oat grain lots (several cultivars) were obtained from various field experiments in southeast Norway (Table 2). Fourteen lots were harvested in 2015 at four different field locations, and ten were harvested in 2018 at one location. The grain lots were chosen based on preliminary tests showing that they contained HT-2 and T-2 toxins, which were the mycotoxins we were most interested in in this study. A sample of approximately 300g of harvested grain (raw material) of each lot was obtained by deviding on a riffle divider (Rationell Kornservice AS, Esbjerg, Denmark). After slight air cleaning (blowing) at “low speed” to remove dust, weed seeds and trash/straw pieces, each sample was further divided into sub-samples of approximately 100 g (unsorted sample) and 200 g. Each of the 200 g samples was size sorted into a large and small kernel fraction by passing the grain through a laboratory scale grain screening machine (in-house made, sieve size = 2.2 mm) at Kimen Seed Laboratory. The weight of unsorted, large and small kernel samples from each grain lot was recorded. Materials of the two size fractions, in addition to the unsorted sample, were ground on a high-speed rotor mill (ZM 200, Retsch, Haan, Germany) fitted with a 1 mm sieve and stored at −20 °C until analyses.

### 4.2. Mycotoxin Analyses

All grain samples were analyzed for the content of eleven different mycotoxins by using LC-MS/MS. The sample preparation was done according to the procedure published by Klötzer and Lauber [51] except that only 5 g aliquot of each sample was extracted with 20 mL mixture of acetonitrile and water (80:20 v/v). The analyses of the mycotoxins detected as cations (HT-2, T-2, Enns, BEA and ZEA) in grain samples harvested in 2015 were carried out using a Waters Ultima Pt MS/MS-detector, whereas the analyses of the mycotoxins detected as anions (DON, NIV, sum of 3-acetyl-DON and 15-acetyl-DON) were performed with a Thermo high resolution accurate mass (HRAM) Q-Exactive Orbitrap instrument. The mycotoxin analysis of grain from 2018 was carried out using HRAM Q-Exactive Orbitrap exclusively, using electrospray polarity switching in order to detect all the ionized mycotoxins in one run (Table 4). The toxins were separated on a Thermo Accucore aQ (100 × 2.1 mm i.d., 2.6 µm) column. A linear mobile phase gradient was used, starting with 100% water in 5 mM ammonium acetate reaching 100% methanol in 5 mM ammonium acetate after 9 min. The total run time was 18 min. The injection volume was 5 µL (Waters instrument) or 1 µL (Thermo instrument), the flow 0.3 mL/min and the column temperature was 30 °C. In the negative mode, mycotoxins were detected as acetate-adducts [M+CH_3_COO]^−^ and in the positive mode mycotoxins were detected as ammonium adducts [M+NH_4_]^+^ or hydrogen adducts [M+H]^+^. The identification criteria were retention time (RT) matched to reference standard, precursor ion accurate m/z mass within 5 ppm accuracy and the presence of at least one targeted product ion with accurate mass within 5 ppm accuracy and produced by fragmentation of the precursor ion. An in-house library of product ion spectra (MS2) for the mycotoxins aided in the identification. Quantification was based on the peak height of the precursor ions. Reference standards of the mycotoxins were purchased from Merck, Darmstadt, Germany. Calibration standards were prepared in the range of 1–1000 µg/kg. Limit of quantification (LOQ) was 1–10 µg/kg. The recovery of HT-2 and T-2 was confirmed to 100% using a certified oat reference material. Recovery of the other toxins was determined from spiked control samples that were prepared with each batch of samples. Recovery was 100% for DON, 3+15-Acetyl-DON and EnnB, 60%–70% for NIV, ZEA, EnnA, EnnA1 and EnnB1, and 45% for BEA. Our method reported the correct levels of HT-2, T-2, DON and ZEA (z-scores lower than 0.35) in oat meal in a proficiency test in 2019 [52].

### 4.3. Data Analyses

The mean, minimum and maximum toxin concentration was calculated for each toxin for both years using Minitab 18. Percentage reductions of toxins in the large grain fractions and percentage increases in the small grain fractions were calculated compared to concentrations in the unsorted grain. Percentage weight reduction was calculated from the weight of the unsorted sample (= 100%) and the weight of the large and small kernel fractions (sum = 100%). The mean toxin levels in the small or the large grain fractions were compared to the mean toxin level in the unsorted grain fraction using a paired *t*-test in Minitab 18. The confidence level was adjusted according to the Bonferroni method to obtain a simultaneous confidence level of 95%, and the differences between the means were considered as significant when *p*-values ≤ 0.05/2. The following relationships were analyzed by linear regression in Minitab 18: (i) the percentage of HT2+T2 reduction vs. the concentration level in the grain lots (unsorted grain), (ii) the sum of toxin concentration (HT2+T2 2015, 2018; DON 2018; EnnB 2015) in the small and the large grain fraction vs. the toxin concentration in the grain lots (unsorted grain), (iii) the percentage weight reduction vs. the percentage HT2+T2 reduction, and (iiii) the percentage of weight reduction vs. the HT2+T2 level in the grain lots. The relationships in (i), (iii) and (iiii) were studied across both years.

## Figures and Tables

**Table 1:** Mean, minimum (min) and maximum (max) mycotoxin concentrations (µg/kg) in unsorted oat grain samples, and large and small grain fractions after size sorting (2.2 mm sieve) and mean and range in percentage change in toxin concentrations in sorted grain fractions (large or small) compared to unsorted grain.

Mycotoxin	Grain Category	2015 (*n* = 14)				2018 (*n* = 10)			
		Mean Toxin Conc. (*p*-Value) ^1^	Min–Max Toxin Conc.	Mean % Change in Toxin Conc. ^2^	Range of % Change in Toxin Conc.	Mean Toxin Conc. (*p*-Value) ^1^	Min–Max Toxin Conc.	Mean % Change in Toxin Conc. ^2^	Range of % Change in Toxin Conc.
HT2+T2	unsorted	745	486–1368			178	92–282		
	large	328 (0.000)	197–552	−56	−24/−76	121 (0.001)	70–187	−32	−2/−66
	small	2775 (0.000)	1149–6427	+272	+66/+840	510 (0.005)	192–804	+187	+87/+470
DON	unsorted	46	1–153			191	100–309		
	large	30 (0.341)	0–178	n.a. ^3^	n.a.	145 (0.000)	89–249	−24	−3/−35
	small	52 (0.811)	0–290	n.a.	n.a.	326 (0.000)	245–514	+71	+42/+145
ADON	unsorted	8	1–35			2.2	1–4		
	large	5 (0.461)	0–30	n.a.	n.a.	1.6 (0.009)	1–4	−27	n.a.
	small	17 (0.435)	0–156	n.a.	n.a.	4.3 (0.000)	3–8	+95	n.a.
NIV	unsorted	23	0–66			6	3–9		
	large	11 (0.035)	0–48	n.a.	n.a.	6 (n.a.)	3–7	n.a.	n.a.
	small	57 (0.164)	0–295	n.a.	n.a.	6 (n.a.)	4–10	n.a.	n.a.
EnnA	unsorted	6	1–27			1	1–2		
	large	4 (0.167)	0–20	n.a.	n.a.	1 (n.a.)	0–2	n.a.	n.a.
	small	16 (0.095)	1–91	n.a.	n.a.	2 (n.a.)	1–4	n.a.	n.a.
EnnA1	unsorted	42	1–221			6	3–11		
	large	24 (0.064)	1–151	n.a.	n.a.	7 (0.588)	2–13	n.a.	n.a.
	small	134 (0.099)	1–896	n.a.	n.a.	12 (0.016)	4–23	+120	−32/+340
EnnB	unsorted	1059	92–5356			15	8−25		
	large	594 (0.018)	48–3064	−44	+2/−63	18 (0.524)	4−43	n.a.	n.a.
	small	2101 (0.008)	37–5319	+98	−60/+568	33 (0.014)	12−67	+120	−10/+500
EnnB1	unsorted	120	6–452			18	9–38		
	large	62 (0.034)	2–296	n.a.	n.a.	19 (0.708)	5–32	n.a.	n.a.
	small	229 (0.043)	2–1001	n.a.	n.a.	34 (0.022)	13–64	+89	−15/+337
BEA	unsorted	7	1–26			3	1–8		
	large	5 (0.083)	1–22	n.a.	n.a.	3 (n.a.)	1–9	n.a.	n.a.
	small	10 (0.154)	1–48	n.a.	n.a.	4 (n.a.)	1–13	n.a.	n.a.
ZEA	unsorted	1	0–2			n.d. ^4^			
	large	2	1–3	n.a.	n.a.	n.d.			
	small	4	2–7	n.a.	n.a.	n.d.			

^1^*p*-value in paired *t*-test where the mean toxin levels of the large or small fraction is compared to the level of the unsorted fraction. *p*-values ≤ 0.05/2 represent a toxin level that is significant different from the unsorted fraction. ^2^ Percentage change in toxin level in large or small grain fractions compared to the unsorted fraction. Reductions in toxin level compared to the unsorted fraction are shown as negative values and increase as positive values. ^3^ n.a.—not analysed due to no significant difference and/or low toxin levels. ^4^ n.d.—not detected.

**Table 2:** Origin (municipality, field number) and cultivars of oat grain lots from 2015 and 2018, and weight proportions (%) of small and large kernel fractions after size sorting (sieve size 2.2 mm).

Harvest Year	Lot Number	Municipality, Field Number	Cultivar	Weight Proportion (%)	
				Small Kernels	Large Kernels
2015	1	Kongsvinger 1	Belinda	18	82
	2	Kongsvinger 2	Belinda	8	92
	3		Belinda	6	94
	4	Kongsvinger 1	Belinda	24	76
	5		Vinger	27	73
	6		Belinda	15	85
	7		Belinda	22	78
	8		Vinger	33	67
	9		Belinda	22	78
	10	Østre Toten	Dovre	11	89
	11		GN12142	9	91
	12	Hamar	Odal	13	87
	13		Nord09/127	9	91
	14		Poseidon	6	94
	Average weight proportion ^1^			15	85
2018	15	Kongsvinger 3	Ringsaker	32	68
	16		GN1311	25	75
	17		Belinda	14	86
	18		Vinger	28	72
	19		Årnes	28	72
	20		Nord13/322	9	91
	21		Gunhild	16	84
	22		GN14182	26	74
	23		GN14209	17	83
	24		GN15154	16	84
	Average weight proportion ^1^			21	79

^1^ Based on fraction weights of all samples.

**Table 3:** Comparison between mycotoxin concentrations (µg/kg) in unsorted grain and the mycotoxin mass balance calculated from the weighted sum of mycotoxins in the large and small kernel fractions and percentage recovery in calculated compared to measured amounts.

Mycotoxin (Harvest Year)	Unsorted Grain (Measured)	Weighted Sum of Large and Small Kernel Fractions (Calculated)	% Recovery (Range)
HT2+T2 (2015)	745	648	87 (59–120)
HT2+T2 (2018)	178	187	105 (75–138)
DON (2018)	191	184	96 (85–117)
EnnB (2015)	1054	820	77 (51–188)

**Table 4:** Parameters for the high resolution accurate mass (HRAM) detection of the analytes including retention time, precursor ion, adducts type of precursor and one of the product ions.

Mycotoxin	Retention Time (min)	Precursor Ion (m/z)	Adduct	Product Ions (m/z)
NIV	3.12	371.13476	[M+CH3COO-]	281.10284
DON	3.83	355.13984	[M+CH3COO-]	295.11835
3+15-Acetyl-DON	5.48	397.15041	[M+CH3COO-]	307.11914
HT-2	7.68	442.24354	[M+NH4+]	363.12781
T-2	7.99	484.25411	[M+NH4+]	305.13818
ZEA	8.71	319.15400	[M+H+]	187.07544
Enn B	9.93	657.44331	[M+H+]	196.13345
Enn B1	10.07	671.45896	[M+H+]	654.43317
BEA	10.09	801.44331	[M+H+]	134.09669
Enn A	10.16	685.47461	[M+H+]	210.14906
Enn A1	10.28	699.49026	[M+H+]	228.15967
